# Healthy lifestyle scores associate with incidence of type 2 diabetes mediated by uric acid

**DOI:** 10.1186/s12986-023-00763-y

**Published:** 2023-11-01

**Authors:** Xinyue He, Wei Shao, Senhai Yu, Jiazhou Yu, Changzhen Huang, Haiqing Ren, Chengguo Liu, Yuying Xu, Yimin Zhu

**Affiliations:** 1 Department of Epidemiology and Biostatistics and Department of Respiratory Diseases of Sir Run Run Show Hospital, Affiliated to School of Medicine, Zhejiang University, Hangzhou, 310058 Zhejiang People’s Republic of China; 2 Zhejiang Putuo Hospital, Zhoushan, Zhejiang People’s Republic of China; 3 Xiaoshan District Yiqiao Community Health Service Center, Hangzhou, Zhejiang People’s Republic of China; 4 Jockey Club School of Public Health and Primary Care, The Chinese University of Hong Kong, Hong Kong Special Administrative Region, People’s Republic of China; 5 Dongyang Traditional Chinese Medicine Hospital, Dongyang, Zhejiang People’s Republic of China; 6 Department of Epidemiology and Biostatistics and Teaching Experiment Center for Public Health, Zhejiang University, Hangzhou, Zhejiang People’s Republic of China

**Keywords:** Cohort study, Healthy lifestyle score, Uric acid, Type 2 diabetes mellitus

## Abstract

**Background:**

Whether and to what extent serum uric acid (SUA) mediates the association between combined lifestyle behaviors and type 2 diabetes mellitus (T2DM) remain unclear. This study aimed to investigate the role of SUA in the relationship between healthy lifestyle scores (HLS) and the incidence of T2DM.

**Methods:**

This prospective study used data from Zhejiang Metabolic Syndrome cohort. A HLS (5-point scale including healthy waist circumference (WC), never smoking, high physical activity, healthy diet and moderate alcohol intake) was estimated in 13,919 participants, who had SUA at baseline examination in 2009–2014, and were followed-up to 2021–2022 to ascertain incident of T2DM. Cox proportional hazards models and mediation analysis were used to examine the associations between HLS, SUA and T2DM.

**Results:**

We included 13,919 participants aged 18 years or older without diabetes at baseline (mean age 54.6 [SD 13.9] years, 58.7% female). During a median follow-up of 9.94 years, 645 cases of T2DM occurred. Compared with participants with a poor HLS, those with 4–5 low-risk lifestyle factors showed a 60% reduction in the risk of developing T2DM (adjusted HR, 0.40; 95% CI: 0.28–0.57). Further, the population-attributable risk percent (95% CI) of T2DM for poor adherence to the overall healthy lifestyle (< 4 low-risk factors) was 43.24% (30.02%, 56.46%). The HLS was inversely associated with SUA level. With per score increased in HLS, the beta (95% CI) of SUA (log transformed) was − 0.03 (− 0.03, − 0.02), and the odds ratio (95% CI) of hyperuricemia was 0.82 (0.77, 0.86). The relationship between the HLS and risk of T2DM was mediated by SUA with a 13.06% mediation effect. There was no significant combined effect of HLS and SUA on risk of T2DM (*P =* 0.097).

**Conclusions:**

The relationship between overall healthy lifestyle behaviors and T2DM was reconfirmed and the association appeared to be mediated by SUA. The mediation effect of baseline SUA was more pronounced among women who were below 60 years old.

**Supplementary Information:**

The online version contains supplementary material available at 10.1186/s12986-023-00763-y.

## Introduction

Type 2 diabetes mellitus is one of the common metabolic diseases in the world. Approximately 536.6 million people worldwide in 2021 are affected by T2DM, and the number is projected to increase to 783.2 million by 2045 [1]. Given the increased health burden related to morbidity and mortality, T2DM has now become a major global health challenge.

It is well documented that lifestyle risk factors such as poor diet, physical inactivity, obesity, smoking, and excessive alcohol drinking cause adverse effects on health outcomes [2–4]. Previous studies have established a healthy lifestyle score to investigate the associations between combined lifestyle factors and health outcomes. Adherence to multiple healthy lifestyle habits has been associated with lower risk of T2DM [5–7], a long-life expectancy free of major chronic diseases [8], and lower risk for mortality [9, 10].

The inverse association of HLS with incident T2DM, is substantially explained by differences in lifestyle-related plasma metabolites measured years before the T2DM clinical diagnosis [7]^.^ SUA is the final product of purine metabolism, and elevated SUA is associated with increased fasting plasma glucose (FPG) in patients with prediabetes [11] and increase risk of new-onset impaired fasting glucose (IFG) and diabetes mellitus [12]. Simultaneously, considerable evidence suggests that people who adhere to a healthy lifestyle, such as high physical activity, no smoking, and healthy dietary pattern, have apparently reduced risk of hyperuricemia [13–15].

However, to our best knowledge, the study to explore the association of joint lifestyle factors with SUA levels is limited. Whether and to what extent SUA mediates the association between combined lifestyle behaviors and T2DM remain unclear. Hence, this study examined the association between combined lifestyle factors, including smoking status, alcohol consumption, diet behavior, physical activity and WC, and risk of T2DM. In addition, we also evaluated the potential mediation effect of SUA in the association between overall lifestyle factors and risk of T2DM.

## Materials and methods

### Study population

The subjects in this study were recruited from the Zhejiang Metabolic Syndrome cohort, a community-based prospective cohort study conducted in Zhejiang province located in Southeast China between 2009 and 2014. Details of the cohort are described elsewhere [16]. The participants were recruited from three counties of Zhejiang, and were followed up until 1 February 2021 and 30 June 2022, respectively.

The participants who were included into the study were aged ≥ 18 years. The exclusion criteria of the subjects for this analysis were as follows [1]: diagnosed with T2DM at baseline; [2] presence of severe chronic diseases at baseline such as cancer, coronary heart disease (CHD), stroke; [3] missing value of lifestyle factors or age; [4] lost to follow-up. Finally, a total of 13,919 participants were included in this study. The detailed participant selection process is shown in Additional file 1: Fig. S1.

### Data collection and assessment

Each participant underwent a face-to-face questionnaire-based interview, clinical health examination, and routine biochemical determination at baseline and first follow-up, as previously reported [16].

Demographic data were collected using a standard structured questionnaire. Variables included age, sex, smoking and alcohol consumption behavior, physical activity, history of T2DM and hypertension, and family history of T2DM, hypertension, CHD, and stroke. Smoking behavior was grouped as current, former, and never. Current smoking was defined as smoking at least one cigarette per day and lasting for more than half a year. Former smoking was defined as stopping smoking for at least half a year [17]. Alcohol drinking was classified into three categories as never, moderate (< 3 times/week) and heavy drinking (≥ 3 times/week) according to frequency on average in the past 12 months. International Physical Activity Questionnaire (IPAQ) was used for the assessment of the average amount of time per week engaged in exercise activities [18]. The energy expended for each activity in metabolic equivalent (MET) hours per week (MET-h/week) was calculated according to the instruction. We defined high physical activity level as the highest sex-specific quartile. Trained interviewers inquired about dietary intake using the semi-quantitative food frequency questionnaire (FFQ). Participants were asked the frequency and consumption of several food on average during the previous year. Each food intake was calculated by the product of the frequency of intake and the amount consumed at each frequency unit. The red meat intake contained pork, beef, and lamb.

Anthropometric data, including weight, height, and waist circumference (WC), were measured by well-trained investigators following a standard protocol. Height and weight were measured using a scale, with the subjects wearing light clothing and without shoes. WC was measured at the midpoint between the iliac crest and lowest rib. Blood pressure was measured in the sitting position with a mercury sphygmomanometer. Systolic blood pressure (SBP) and diastolic blood pressure (DBP)were recorded as the average of three repeated measurements with 30-s intervals. Hypertension was defined as SBP ≥ 140 mmHg or DBP ≥ 90 mmHg or diagnosis with hypertension by their doctors or current use of antihypertensive medication according to the National High Blood Pressure Education Program [19].

Biochemical data were collected from each subject after a 12-h overnight fast. Biochemical variables, including SUA, triglycerides (TG), total cholesterol (TC), high-density lipoprotein cholesterol (HDL-C), and low-density lipoprotein cholesterol (LDL-C), were determined using biochemical auto-analyzer (Hitachi 7060, Tokyo, Japan). Fasting plasma glucose (FPG) was analyzed by the glucose oxidase method with a Beckman Glucose Analyzer (Beckman Instruments, Irvine, CA, USA).

### Determination of healthy lifestyle scores (HLS)

Five lifestyle factors were included in this study based on previous studies and Dietary Guidelines for Chinese Residents: smoking, alcohol intake, physical activity, dietary habits, and body composition [20, 21]. In our study, never smoking was defined low-risk, compared with current or past smoking. Never and moderate drinking was defined as low-risk for alcohol intake. Based on the calculated MET-h/week, high physical activity level, defined as highest sex-specific quartile, was considered low-risk. For dietary habits, we generated a dietary score ranging from 0 to 3 based on three components: fruits, vegetables and red meat. Healthy dietary habit was considered low-risk and was defined as meeting at least two ideal diet components, classified as the highest quartile consumption of fruits and vegetables and the lowest quartile consumption of red meat. For body composition, low-risk WC was defined as < 90 cm for male and < 85 cm for female, which was indicator for abdominal obesity [22]. Finally, we constructed the overall HLS by summing the numbers of present low-risk lifestyle behaviors in each participant. The score ranged from 0 to 5, with higher score indicating healthier lifestyle.

### Definition of T2DM and hyperuricemia

The incidence of T2DM was identified through the local chronic disease surveillance system. According to the criteria of the American Diabetes Association [23], participants were considered to have T2DM when meeting at least one of the following criteria: (1) FPG ≥ 7.0 mmol/L (126 mg/dL); (2) self-reported T2DM diagnosed by a physician; or (3) currently taking antidiabetic medication. Hyperuricemia at baseline was SUA ≥ 420 µmol/L in males and SUA ≥ 360 µmol/L in females [24].

### Statistical analyses

Participants were divided into four groups according to HLS (0–1, 2, 3, 4–5) to ensure statistical power. Continuous variables with normal distribution were described as means and standard deviations (SD), while continuous variables with skewed distribution were presented as median (interquartile range). Categorical variables were described as number (percentages). Comparisons of the characteristics among the four HLS groups were performed using one-way ANOVA for continuous variables, and chi-square test or Fisher exact test for categorical variables. Person-years of follow-up were calculated from the date at baseline to the first occurrence of T2DM, mortality, or the end of the study, whichever came first. Incidence of T2DM was calculated as the number of incident cases divided by the total person-years of follow-up.

Multiple Cox regression model was used to calculate the hazard ratio (HR) and its confidence interval (95% CI) to examine the association between HLS and the risk of T2DM in the following two ways: (1) as a categorical variable (0–1, 2, 3, 4–5 points), with the lowest HLS group as reference group; (2) as a continuous variable (per point increase). The proportional hazard assumption was tested using the Schoenfeld residual, and the results showed no violation on the assumptions. To adjust for the potential confounding bias and to examine the consistency of the results, we constructed two sets of models: Model 1 adjusted for age, sex, family history of T2DM, family history of hypertension, prevalence of hypertension, use of antihypertensive medication, and use of lipid-lowing medication; Model 2 additionally adjusted for TG, HDL-C, FPG. Further,. We used restricted cubic splines with four knots at the 5th, 35th, 65th, and 95th centiles to flexibly model the dose–response relationships of HLS with T2DM with a smoothly joined piecewise polynomial, and test for potential non-linearity by using a likelihood ratio [25]. We also calculated the population-attributable risk percent (PAR%) to estimate the proportion of T2DM that could theoretically be avoided if all participants had adhered to four or more low-risk lifestyle behaviors [26].

Once the temporal relationships of HLS with T2DM was established, we performed a series of analyses to examine whether the association between HLS (X) and T2DM (Y) were mediated by baseline SUA levels or hyperuricemia (M): (1) the association between HLS (X) and baseline SUA levels (continuous, nature log transformed) and hyperuricemia (M) was examined using linear regression model or logistic regression model, with all the covariates adjusted; (2) the associations between baseline SUA levels or hyperuricemia (M) and T2DM (Y) were examined by three sets of Cox proportional hazard models with all the covariates adjusted: (a) SUA level as a categorical variable based on sex-specific quartiles (cut-points were 312, 363 and 423 µmol/L in males, and 231, 271 and 317 µmol/L in females), with the lowest quartile group as reference group; (b) SUA level as a continuous variable (per SD increase); and (c) hyperuricemia as a dichotomous variable; (3) the mediation effect of baseline SUA levels or hyperuricemia (M) in the association between HLS (X) and T2DM (Y) was examined by causal mediation analysis using the model-based approach [27, 28], adjusted for age, sex, family history of hypertension, use of antihypertensive medication, and use of lipid-lowing medication, which were sufficient to control for exposure-outcome, mediator-outcome, and exposure-mediator confounding [29, 30]. A bootstrap analysis with 1,000 replications was applied to compute the CIs of the proportions of mediations. The linear trend test for HLS was performed by treating the number of low-risk factors as a continuous variable; for SUA by assigning the sex-specific median to each category and then modeling this as a continuous variable in the model. To evaluate whether there was a combined effect of HLS and SUA on T2DM, we further stratified the analyses by hyperuricemia status and HLS. The interaction effect between HLS and SUA was assessed by including interaction terms in the multivariable model and tested with the likelihood ratio test.

Subgroup analyses were conducted by stratification according to sex (male or female) and age (< 60 or ≥ 60 years). To test the robustness of our results, we performed mediation analysis that additionally adjusted for baseline FPG. In addition, we generated the new HLS without low-risk drinking behavior, due to lack of amount of consumption which may involve inaccurate classification, and reevaluated the association of HLS and T2DM.

All of the statistical analyses were performed using R software version 4.0.3. All P values were two-sided, and *P <* 0.05 was defined as statistically significant. The borderline significance was defined as the P > 0.05, but < 0.10 (0.05 ≤ *P <* 0.10).

## Results

### Baseline characteristics

Baseline characteristics of the study sample according to HLS are summarized in Table 1. Overall, the mean age was 54.6 years (SD 13.9) and 58.7% of the sample were females. Moreover, 2775 (19.9%) individuals had an ideal HLS (4–5 points), and 1248 (9.0%) individuals had a poor HLS (0–1 points). Participants with lower HLS were more likely to be older, with higher levels of SPB, DPB, SUA, FPG, TG, and with lower levels of HDL-C.

**Table 1:** Baseline characteristics of the study population according to HLS^#^

Variables	Overall	HLS				*P* value
		0–1	2	3	4–5	
N	13,919	1248	3795	6101	2775	–
Age, year (mean (SD))	54.6 (13.9)	57.7 (12.3)	57.4 (13.0)	53.5(14.5)	51.7 (13.5)	< 0.001
Female (n, %)	8171 (58.7)	122(9.8)	1580 (41.6)	4202 (68.9)	2267 (81.7)	< 0.001
Smoking (n, %)	3951 (28.4)	1158 (92.8)	1929 (50.8)	809 (13.3)	55(2.0)	< 0.001
Heavy drinking (n, %)	1561 (11.2)	872 (69.9)	540 (14.2)	139 (2.3)	10 (0.4)	< 0.001
High physical activity (n, %)	3500 (25.1)	56 (4.5)	350 (9.2)	1126 (18.5)	1968 (70.9)	< 0.001
Healthy diet (n, %)	1864 (13.4)	20 (1.6)	123 (3.2)	520 (8.5)	1201 (43.3)	< 0.001
WC, cm (mean (SD))	79.93 (9.51)	88.12 (9.05)	84.69 (9.36)	77.39 (8.26)	75.31 (7.06)	< 0.001
SBP, mmHg (mean (SD))	130.59 (21.89)	138.13 (21.09)	134.02 (22.02)	128.87 (21.83)	126.32 (20.64)	< 0.001
DBP, mmHg (mean (SD))	78.87 (12.61)	83.47 (12.57)	80.21 (12.77)	77.76 (12.46)	77.44 (12.05)	< 0.001
SUA levels, µmol/L (mean (SD))	317.44 (90.89)	380.48 (89.53)	343.27 (90.89)	302.18 (85.21)	284.84 (78.96)	< 0.001
FPG, mmol/L (mean (SD))	4.92 (0.69)	5.09 (0.72)	4.92 (0.71)	4.89 (0.67)	4.92 (0.67)	< 0.001
TG, mmol/L (median [IQR])	1.23 [0.87, 1.81]	1.35 [0.95, 2.05]	1.35 [0.95, 2.01]	1.19 [0.85, 1.73]	1.13 [0.82, 1.65]	< 0.001
HDL.C, mmol/L (mean (SD))	1.42 (0.37)	1.39 (0.38)	1.37 (0.36)	1.44 (0.36)	1.48 (0.38)	< 0.001
Hypertension (n, %)	5401 (39.5)	682 (55.5)	1712 (45.9)	2121 (35.4)	886 (32.6)	< 0.001
Family history of hypertension (n, %)	3997 (28.7)	452 (36.2)	1007 (26.5)	1715 (28.1)	823 (29.7)	< 0.001
Family history of diabetes (n, %)	713 (5.1)	70 (5.6)	170 (4.5)	313 (5.1)	160 (5.8)	0.102
Use of anti-hypertensive medication (n, %)	1002 (7.2)	136 (10.9)	352 (9.3)	387 (6.3)	127 (4.6)	< 0.001
Use of lipid-lowing medication (n, %)	107 (0.8)	15 (1.2)	34 (0.9)	40 (0.7)	18 (0.6)	0.143

^#^HLS: healthy lifestyle scores, numbers of healthy lifestyle behaviors including healthy WC (< 90 cm for men, or < 85 cm for women), never smoking, high physical activity (top sex-specific quartile of MET-h/week, cut-off values are 321.9 MET-h/week for male and 243.6 for female.), healthy diet (≥ 2 dietary components at ideal levels), and moderate alcohol intake (< 3 times/week)

### Associations between HLS and T2DM risk

During a total of 134,076 person-years (median follow-up period 9.94 years), 645 participants (230 male, 415 female) were diagnosed with T2DM. The association between individual lifestyle factors and the risk of T2DM after adjusting for other lifestyles is shown in Additional file 1: Table S1. WC less than 90 cm in male or less than 85 cm in female, and ≥ 2 ideal components of dietary score, were individually associated with a lower risk of incident T2DM. When healthy lifestyle behaviors were considered jointly, a dose–response relationship of the HLS with risk of T2DM was observed (Table 2, Additional file 1: Fig. S2).

**Table 2:** Adjusted HR for association between HLS and T2DM

HLS	Number of T2DM	Person-year	Incidence per 1000 person-years	Model 1		Model 2	
				HR (95% CI)	*P*	HR (95% CI)	*P*
0–1	82	11,972.06	6.85	1.00	–	1.00	–
2	217	36,149.31	6.00	0.77 (0.59, 1.01)	0.051	0.84 (0.64, 1.10)	0.255
3	271	58,572.80	4.63	0.61 (0.47, 0.81)	< 0.001	0.73 (0.55, 0.96)	0.026
4–5	75	27,382.26	2.74	0.36 (0.26, 0.51)	< 0.001	0.40 (0.28, 0.57)	< 0.001
P-trend	–	–	–	–	< 0.001	–	< 0.001
Per score	–	–	–	0.74 (0.67, 0.81)	< 0.001	0.77 (0.71, 0.85)	< 0.001
PAR% (%, 95% CI) ^#^	–	–	–	–	–	43.24 (30.02,56.46)	–

Model 1: adjusted for age, sex, family history of diabetes, family history of hypertension, prevalence of hypertension, use of anti-hypertensive medication, and use of lipid-lowing medication

Model 2: Model1 + TG (mmol/L), HDL-C (mmol/L), and FPG (mmol/L)

^#^PAR% was calculated to estimate the percentage of T2DM that would have been prevented if all participants had been in more than 4 healthy lifestyle behaviors

HR, hazard ratio; HLS, healthy lifestyle scores; T2DM, type 2 diabetes mellitus; CI, confidence interval; PAR%, population-attributable risk percent

The risk of T2DM decreased dramatically with increasing number of the healthy lifestyle behaviors (*P* for trend < 0.001). After adjusted for age, sex, family history of diabetes and hypertension, prevalence of hypertension, use of antihypertensive and lipid-lowing medication, compared with participants with a one or less low-risk lifestyle factors, those with 3 factors had 39% lower risk of incident T2DM (HR, 0.61; 95% CI 0.47–0.81), and those with 4–5 factors showed a 64% reduction in the risk of developing T2DM (HR, 0.36; 95% CI 0.26–0.51). Moreover, when treating HLS as a continuous variable, T2DM risk decreased by 26% with per point increase in HLS (HR, 0.74; 95% CI 0.67–0.81). Additional adjustment for TG, HDL-C, and FPG, at baseline did not materially alter the association (for 3 factors: HR, 0.73; 95% CI 0.55–0.96; for 4–5 factors: HR, 0.40; 95% CI 0.28–0.57). The PAR% of nonadherence to four or more low-risk lifestyle factors was 43.24% (95% CI 30.02–56.46%). In addition, the estimated PAR% was 25.40% (95% CI 7.04%, 43.77%) for dietary score and 18.54% (95% CI 12.94%, 24.14%) for WC. The consistent association was still observed after excluding the alcohol drinking factor (per score increase adjusted HR, 0.70; 95% CI 0.63–0.77) (Additional file 1: Table S2).

### Associations between baseline SUA level and T2DM risk

The population was classified into four groups (Q1-Q4) according to quartiles of baseline SUA levels: 74-, 312-, 363-, 423–768 µmol/L for male, and 74-, 231-, 271-, 317–859 µmol/L for female. The results of Cox proportional hazard model were shown in Table 3.

**Table 3:** Adjusted HR for association between baseline SUA and T2DM

Group^#^	Number of T2DM	Person-year	Incidence per 1000 person-years	Model 1		Model 2	
				HR (95% CI)	*P*	HR (95% CI)	*P*
Q1	117	31,373.42	3.73	1.00	–	1.00	–
Q2	126	31,590.78	3.99	0.97 (0.76, 1.25)	0.81	0.90 (0.70, 1.16)	0.40
Q3	146	31,395.91	4.65	1.05 (0.82, 1.34)	0.72	0.95 (0.75, 1.22)	0.66
Q4	229	29,984.09	7.64	1.56 (1.24, 1.96)	< 0.001	1.35 (1.07, 1.70)	0.011
P-trend	–	–	–	–	< 0.001	-	0.001
Per SD	–	–	–	1.26 (1.15,1.37)	< 0.001	1.18 (1.08, 1.29)	< 0.001
Hyperuricemia^*^	164	21,109.42	7.77	1.61 (1.34,1.94)	< 0.001	1.43 (1.19, 1.72)	0.001

Model 1: adjusted for age, sex, healthy lifestyle scores (continuous), family history of diabetes, family history of hypertension, prevalence of hypertension, use of anti-hypertensive medication, and use of lipid-lowing medication

Model 2: Model1 + TG (mmol/L), HDL-C (mmol/L), and FPG (mmol/L)

^#^sex-specific quartile of baseline SUA:74-, 312-, 363-, 423–768 µmol/L for male, 74-, 231-, 271-, 317–859 µmol/L for female

^*^Hyperuricemia was defined as SUA ≥ 420 µmol/L for men and SUA ≥ 360 µmol/L for women

HR, hazard ratio; SUA, serum uric acid; T2DM, type 2 diabetes mellitus; CI, confidence interval; SD, standard deviation

Compared with participants at the lowest quartile of SUA, those at the highest quartiles had a 35% (95% CI 1.07–1.70) higher risk of T2DM, after adjusted for all covariates mentioned above. Per SD increasement in SUA was associated with a 18% higher risk of T2DM (95% CI 1.08–1.29). There was also a statistically significant association between hyperuricemia and T2DM risk. Compared with participants without hyperuricemia, those with hyperuricemia had a 43% higher risk of T2DM (95% CI 1.19–1.72).

### Associations between HLS and SUA

The results of regression models for the association between baseline SUA and HLS (Table 4) showed that participants with healthier healthy lifestyles had significantly lower SUA levels.

**Table 4:** Linear or logistic regression models for the association between HLS and SUA

HLS	SUA^#^ (µmol/L)				Hyperuricemia^†^			
	Model 1		Model 2		Model 1		Model 2	
	β (95% CI)	*P*	β (95% CI)	*P*	OR (95% CI)	*P*	OR (95% CI)	*P*
0–1	1.00	–	1.00	–	1.00	–	1.00	–
2	− 0.03 (− 0.04, − 0.01)	< 0.001	− 0.02 (− 0.03, − 0.01)	0.021	0.82 (0.71, 0.95)	0.009	0.88 (0.76, 1.04)	0.126
3	− 0.08 (− 0.11, − 0.07)	< 0.001	− 0.06 (− 0.07, − 0.04)	< 0.001	0.57 (0.49, 0.67)	< 0.001	0.71 (0.60, 0.83)	< 0.001
4–5	− 0.11 (− 0.13, − 0.09)	< 0.001	− 0.08 (− 0.10, − 0.06)	< 0.001	0.45 (0.37, 0.55)	< 0.001	0.55 (0.45, 0.67)	< 0.001
*P*-trend	–	< 0.001	-	< 0.001	–	< 0.001	–	< 0.001
Per score	− 0.04 (− 0.04, − 0.03)	< 0.001	− 0.03 (− 0.03, − 0.02)	< 0.001	0.74 (0.70, 0.78)	< 0.001	0.82 (0.77, 0.86)	< 0.001

Model 1: adjusted for age, sex

Model 2: Model1 + family history of diabetes, family history of hypertension, prevalence of hypertension, use of anti-hypertensive medication, use of lipid-lowing medication, TG (mmol/L), HDL-C (mmol/L), and FPG (mmol/L)

^#^The levels of SUA were nature log transformed

^†^Hyperuricemia was defined as SUA ≥ 420 µmol/L for men and SUA ≥ 360 µmol/L for women

HLS, healthy lifestyle scores; SUA, serum uric acid; CI, confidence interval; OR, odds ratio

Compared with participants with 0 to 1 low-risk lifestyle behavior, HLS was negatively associated with SUA levels (per point increase in HLS: β = − 0.03, *p <* 0.001) and with hyperuricemia (per point increase in HLS: OR = 0.82, *p <* 0.001), after adjusted for age, sex, family history of diabetes and hypertension, prevalence of hypertension, use of antihypertensive or lipid-lowing medication, TG, HDL-C and FPG.

### Mediation effect of SUA in the association of HLS with T2DM risk

**Fig. 1:**
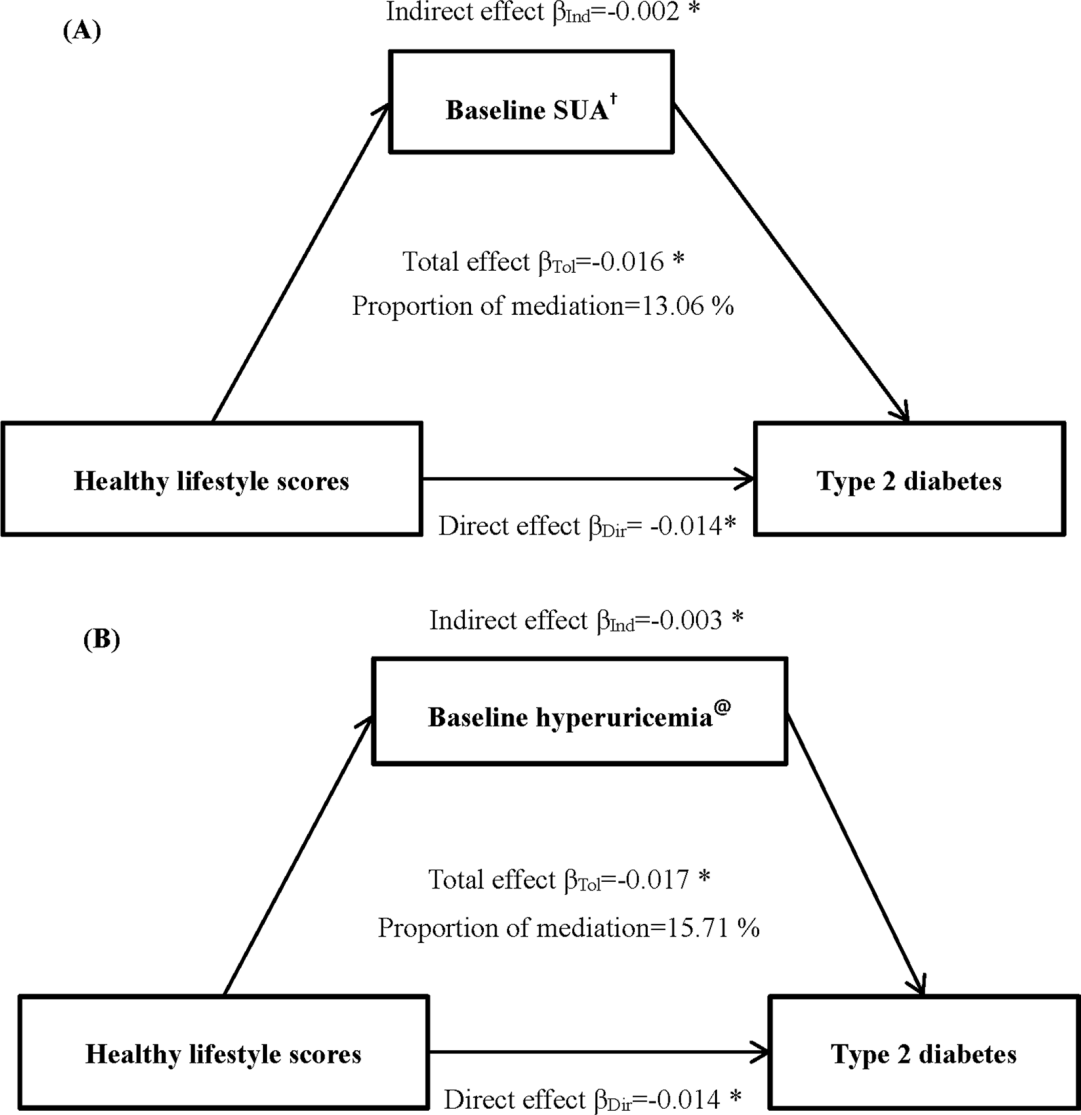
Mediation effect of (**A**) baseline SUA, (**B**) baseline hyperuricemia on the association of healthy lifestyle scores and T2DM. *represent coefficients different from 0, *P <* 0.001. †The levels of SUA were nature log-transformed. @Hyperuricemia was defined as SUA ≥ 420 µmol/L for men and SUA ≥ 360 µmol/L for women

There was no significant combined effect of HLS and SUA on risk of T2DM (*P =* 0.097, Additional file 1: Fig. S3). The mediation analysis (Table 5, Fig. 1) showed that the association of HLS with risk of T2DM was partially mediated by baseline SUA levels (indirect effect: β = − 0.002, *p <* 0.001, proportion of mediation = 14.28%) after adjusted for age, sex, family history of hypertension, use of anti-hypertensive medication and use of lipid-lowing medication. Similar mediation effect was for hyperuricemia (indirect effect: β = − 0.003, *p <* 0.001, proportion of mediation = 15.71%).

**Table 5:** Association of HLS with T2DM mediated by SUA

Effects	SUA^#^ (µmol/L)				Hyperuricemia^†^			
	Model 1		Model 2		Model 1		Model 2	
	β (95% CI)	*P*	β (95% CI)	*P*	β (95% CI)	*P*	β (95% CI)	*P*
Total effects	− 0.022 (− 0.033, − 0.013)	< 0.001	− 0.016 (− 0.021, − 0.009)	< 0.001	− 0.024 (− 0.034, − 0.014)	< 0.001	− 0.017 (− 0.028, − 0.010)	< 0.001
Direct effects	− 0.019 (− 0.028, − 0.010)	< 0.001	− 0.014 (− 0.025, − 0.010)	< 0.001	− 0.020 (− 0.029, − 0.011)	< 0.001	− 0.014 (− 0.024, − 0.009)	< 0.001
Indirect effects	− 0.003 (− 0.006, − 0.001)	< 0.001	− 0.002 (− 0.004, − 0.002)	< 0.001	− 0.004 (− 0.005, − 0.002)	< 0.001	− 0.003 (− 0.004, − 0.001)	< 0.001
Proportion of mediation (%) ^*^	14.38 (5.28, 26.01)	< 0.001	13.06 (6.29, 30.22)	< 0.001	16.07 (9.87, 27.18)	< 0.001	15.71 (6.69, 33.12)	< 0.001

Model 1: unadjusted model

Model 2: age, sex, family history of hypertension, use of anti-hypertensive medication, and use of lipid-lowing medication

^#^The levels of SUA were nature log-transformed

^†^Hyperuricemia was defined as SUA ≥ 420 µmol/L for men and SUA ≥ 360 µmol/L for women

^*^1000 bootstrap resampling

HLS, healthy lifestyle scores; T2DM, type 2 diabetes mellitus; SUA, serum uric acid; CI, confidence interval

**Fig. 2:**
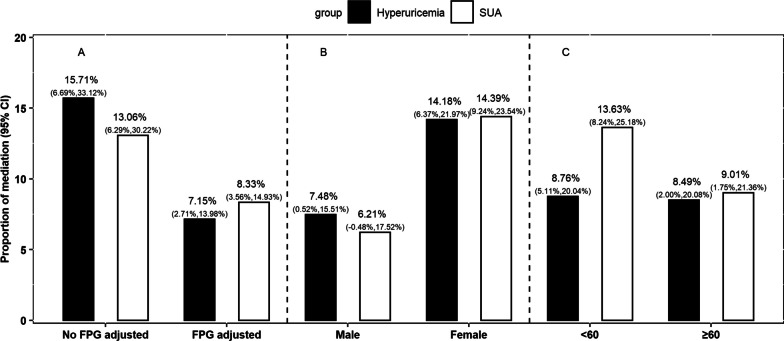
Mediation effect of baseline SUA or hyperuricemia on the association of HLS and T2DM: (**A**) additionally adjustment for FPG or not, (**B**) stratified by sex, (**C**) stratified by age(years), respectively. The levels of SUA were nature log-transformed. Hyperuricemia was defined as SUA ≥ 420 µmol/L for men and SUA ≥ 360 µmol/L for women. HLS, healthy lifestyle scores; T2DM, type 2 diabetes mellitus; SUA, serum uric acid

After adjusting for FPG, the proportion of mediation was reduced, indicating that FPG might play a role between SUA and T2DM (Fig. 2, Additional file 1: Table S3). The analysis after stratified by sex and age found that the mediation effect of SUA between HLS and risk of T2DM was more pronounced among females and individuals aged below 60 years (Fig. 2, Additional file 1: Table S4, Additional file 1: Table S5).

## Discussion

In this population-based cohort study with 9.94-year follow-up, the HLS, a composite healthy lifestyle measure of smoking, alcohol consumption, physical activity, diet, and body composition, was inversely associated with the risk of T2DM, and such association was partially mediated by the level of SUA at baseline. For adoption of each additional low-risk lifestyle behavior, the risk of T2DM decreases by 23%, and 43% of T2DM could have been avoided if the population possess 4–5 healthy lifestyle behaviors. Individuals with more healthy lifestyle behaviors are more likely to have lower level of SUA, which partially contributes to the lower risk of T2DM.

There has been preponderant evidence on the impact of individual lifestyle behaviors. For example, there is evidence that SUA is linked to dietary factors [31, 32], and adiposity and alcohol consumption are associated with a higher SUA [33]. Mean change in BMI, but not in WC, was statistically significantly associated with change in SUA over a 1-year follow-up in males [34]. For smoking, consensus has yet to be reached regarding its effect on serum urate levels [35, 36]. To date, few studies have attempted to examine the association between combined healthy lifestyle and SUA. Our study provided novel evidence that the SUA level decreases with increase in healthy lifestyle behaviors. Lifestyle interventions can reshape the gut microbiota [37]. *Faecalibacterium* [38] and *Clostridium* [39], as an intestinal microbial preparation, can produce butyric acid by fermenting dietary fiber in the intestine. Intestinal butyric acid amounts were related to uric acid metabolism in humans [40]. *Escherichia-Shigella* could secrete xanthine deaminase, which can convert hypoxanthine and xanthine into uric acid [41].

Our mediation analyses revealed that uric acid in plasma may be a potential pathway of mechanism in the inverse association between combined lifestyle behavior and risk of T2DM. Previous studies have revealed the mediation effect of SUA [42], and obesity [43] in the association between individual lifestyle factors and risk of T2DM. There is also evidence on the role of plasma metabolites in explaining the association of overall lifestyle and incident T2DM [7]. The strong association between HLS with metabolites profiles reflected several metabolic pathways. And most of the identified HLS-related metabolites were also prospectively associated to T2DM [7]. The mediation mechanism of SUA between HLS and risk of T2DM could be associated with gut microbiota [40, 44]. Moreover, the mediation effect of SUA in inverse association between HLS and risk of T2DM was greater among females and those aged below 60. It’s because gut microbiota could affect material metabolism in sex and age differences [45–47]. According to our study, women who were below 60 years old should pay more attention to healthy lifestyle behavior to stable their SUA levels so that the prevention of T2DM.

The major strengths of this study are the prospective cohort study design and long follow-up period. To the best of our knowledge, this is the first study to investigate the mediating role of SUA in the relationship between combined healthy lifestyle and diabetes. There are also several limitations to our study. First, this study cannot establish causal relationship between HLS and SUA due to their data both from baseline. Future studies with repeatedly measured data are required to replicate our findings. Second, the information on lifestyle factors, except for WC, was self-reported, which may introduce information bias. However, the standard structured questionnaire and well-trained investigators increased the accuracy and reliability of the data. Third, although this simple additive method to develop HLS has been widely used, the underlying assumption is that the associations between different lifestyle factors and outcomes are the same, which may not be true. It’s necessary to generate a weighted HLS and examined the associations in a larger representative population. In addition, low-risk alcohol consumption was classified based on frequency rather than amount of consumption, which may involve misclassification. However, the inverse association between HLS and T2DM was still observed after excluding the alcohol drinking factor. Finally, we did not adjust for socioeconomic status in the analysis, which may raise concerns of unmeasured confounding. Several residual confounding could not be excluded due to the observational study design. Clinical trials and gut microbiota are definitely needed to verify the role of SUA in the association of lifestyle and T2DM.

## Conclusions

Our findings suggest that adherence to overall healthy lifestyle behaviors was associated with a lower risk of T2DM and support the importance of programs and interventions targeting at promoting health behaviors to reduce the risk of T2DM. In addition, our data showed significant partial mediation effect of SUA in the associations between lifestyle behaviors and T2DM. Further studies are warranted to confirm our findings.

### Supplementary Information

**Additional file 1: Figure S1** Flowchart of participants included in the study. **Figure S2** Dose–response relationship of HLS with risk of T2DM. **Figure S3** The combined effect of HLS and hyperuricemia on T2DM. **Table S1**. Adjusted HR for association between single healthy lifestyle behavior and T2DM. **Table S2**. Adjusted HR of T2DM according to new HLS without alcohol behavior. **Table S3**. Association of HLS with T2DM mediated by SUA after additionally adjusting for FPG. **Table S4**. Association of HLS with T2DM mediated by SUA stratified by sex. **Table S5**. Association of HLS with T2DM mediated by SUA stratified by age.

## Data Availability

The datasets used during the current study are available from the corresponding author on reasonable request.

## Abbreviations

SUA: Serum uric acid
T2DM: Type 2 diabetes mellitus
HLS: Healthy lifestyle scores
WC: Waist circumference
FPG: Fasting plasma glucose
IFG: Impaired fasting glucose
CHD: Coronary heart disease
IPAQ: International Physical Activity Questionnaire
FFQ: Food frequency questionnaire
SBP: Systolic blood pressure
DBP: Diastolic blood pressure
BMI: Body mass index
TG: Triglycerides
TC: Total cholesterol
HDL-C: High-density lipoprotein cholesterol
LDL-C: Low-density lipoprotein cholesterol
SD: Standard deviations
HR: Hazard ratio
CI: Confidence interval
PAR%: Population-attributable risk percent
