# Supplementary material for: Healthy lifestyle scores associate with incidence of type 2 diabetes mediated by uric acid
Source: Nutr Metab (Lond). 2023 Nov 1;20:47. doi: 10.1186/s12986-023-00763-y (PMC10619235; doi:10.1186/s12986-023-00763-y)
Supplement: Supplementary file 1 — Additional file 1: Figure S1 Flowchart of participants included in the study. Figure S2 Dose–response relationship of HLS with risk of T2DM. Figure S3 The combined effect of HLS and hyperuricemia on T2DM. Table S1. Adjusted HR for association between single healthy lifestyle behavior and T2DM. Table S2. Adjusted HR of T2DM according to new HLS without alcohol behavior. Table S3. Association of HLS with T2DM mediated by SUA after additionally adjusting for FPG. Table S4. Association of HLS with T2DM mediated by SUA stratified by sex. Table S5. Association of HLS with T2DM mediated by SUA stratified by age. [file 12986_2023_763_MOESM1_ESM.docx]

Exclude 27 subjects loss to follow-up for diabetes

Exclude subjects with CVD (n=557), cancers (n=236) and diabetes (n=1,383) at baseline

22,141 subjects who participated in the baseline surveys (between 2009-2014)

10129

17,247 subjects aged ≥45 years from CHARLS (Wave 1)

9,432 subjects aged ≥50 years from ELSA (Wave 2)

19,965 healthy subjects

Exclude subjects with missing data of lifestyle factors (n=5,941), age (n=78) at baseline

13,946 subjects with complete lifestyle behaviors

**Figure S1. Flow diagram of participants included in the study.**

13,919 eligible subjects included at baseline

**FigureS2 Dose-response relationship of HLS with risk of T2DM.** X-axis showed the numbers of low-risk lifestyle behaviors, and y-axis showed the HR of T2DM. Multivariable- adjusted models were adjusted for all potential confounding factors mentioned above. HR, hazard ratio; HLS, healthy lifestyle scores; T2DM, type 2 diabetes mellitus; CI, confidence interval.


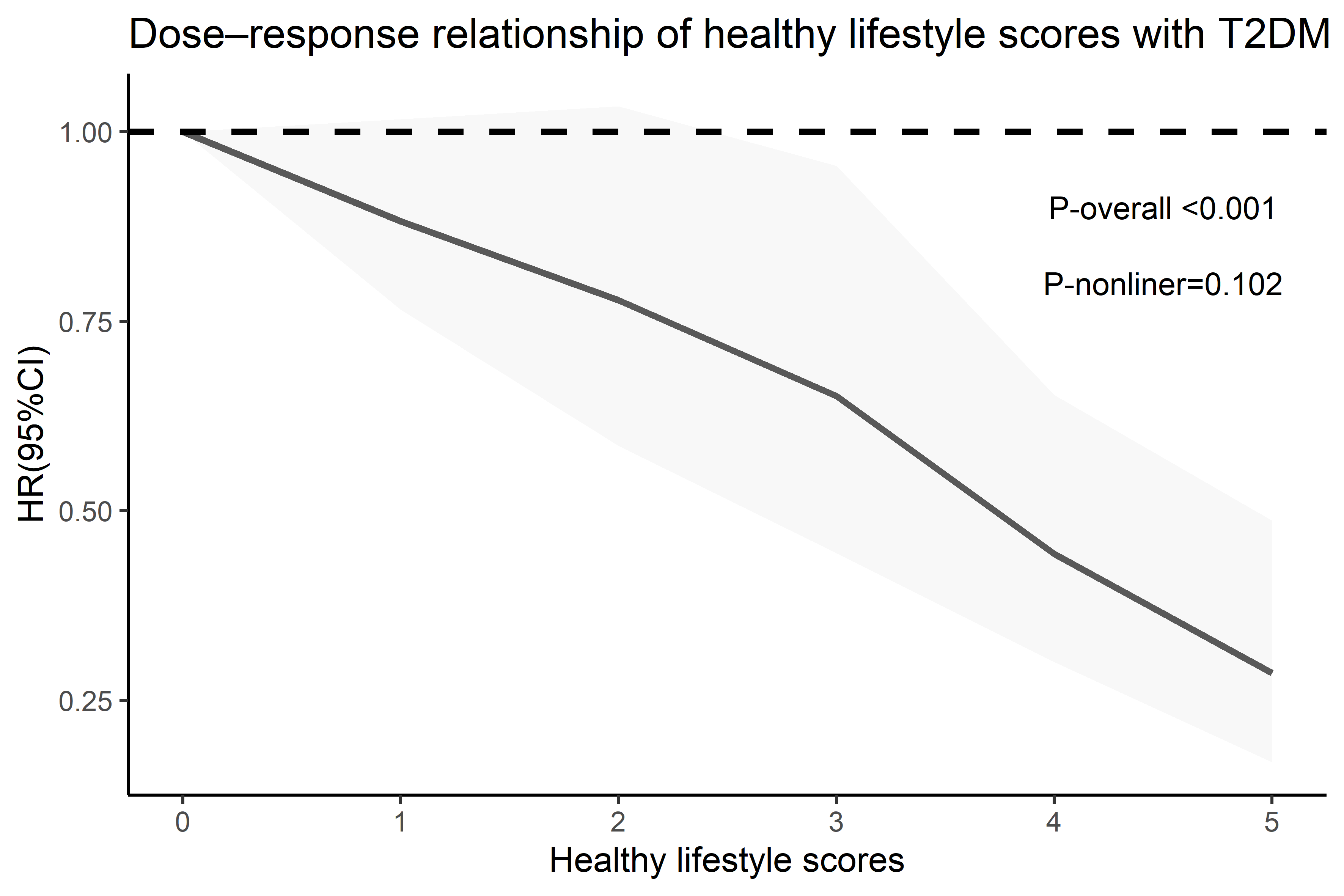


**FigureS3 The combined effect of HLS and hyperuricemia on T2DM.** Low HLS was defined as HLS < 4. Hyperuricemia was defined as SUA ≥ 420 µmol/L for men and SUA ≥ 360 µmol/L for women. The model was adjusted for all potential confounding factors mentioned above. *P* for interaction between HLS and SUA was 0.097. HR, hazard ratio; HLS, healthy lifestyle scores; T2DM, type 2 diabetes mellitus; CI, confidence interval.


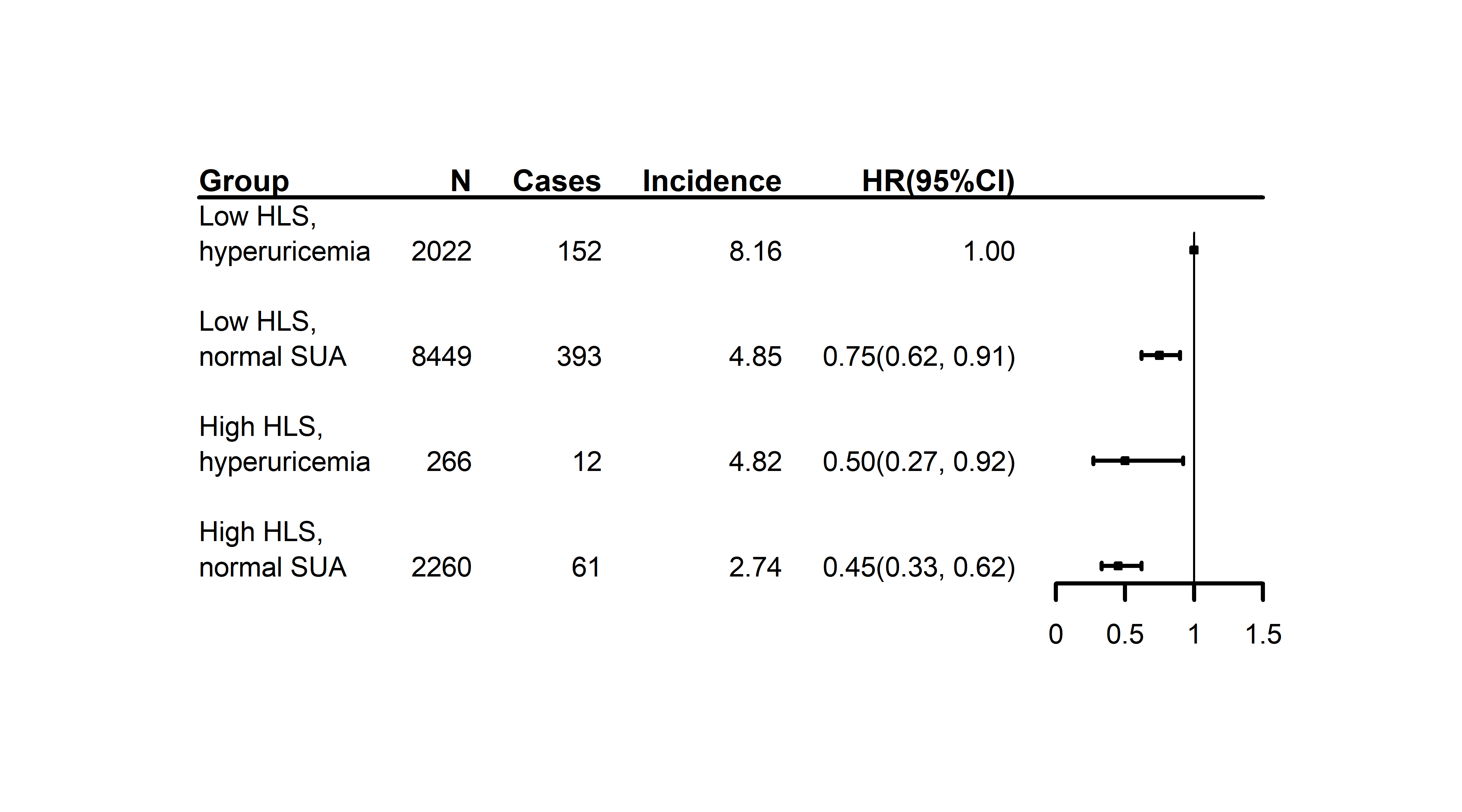


| **Table S1 Adjusted HR for association between single healthy lifestyle behavior and T2DM** | | | | | | | | |  |
| --- | --- | --- | --- | --- | --- | --- | --- | --- | --- |
| Lifestyle behaviors | Number of T2DM | Person-years | Incidence per 1000 person-years | Model 1 | |  | Model 2 | | PAR% (%, 95% CI) ^*^ |
|  |  |  |  | HR (95% CI) | *P* |  | HR (95% CI) | *P* |  |
| **Waist circumference (cm)** |  |  |  |  |  |  |  |  | 18.54 (12.94,24.14) |
| ≥90 (men) or ≥85 (women) | 297 | 31422.84 | 9.45 | 1.00 |  |  | 1.00 |  |  |
| < 90 (men) or < 85 (women) | 348 | 102653.58 | 3.39 | 0.37 (0.32, 0.43) | <0.001 |  | 0.55 (0.47, 0.65) | <0.001 |  |
| **Smoking status** |  |  |  |  |  |  |  |  | / |
| Current or ever smoker | 161 | 37657.21 | 4.28 | 1.00 |  |  | 1.00 |  |  |
| Never smoker | 484 | 96419.21 | 5.02 | 1.12 (0.93, 1.36) | 0.226 |  | 0.90 (0.71, 1.15) | 0.405 |  |
| **Alcohol intake (****times/week)** |  |  |  |  |  |  |  |  | / |
| ≥ 3 | 75 | 15216.27 | 4.93 | 1.00 |  |  | 1.00 |  |  |
| < 3 | 570 | 118860.15 | 4.80 | 0.99 (0.76, 1.27) | 0.913 |  | 1.26 (0.97, 1.66) | 0.085 |  |
| **Physical activity** |  |  |  |  |  |  |  |  | / |
| Bottom three quartiles | 502 | 99726.95 | 5.03 | 1.00 |  |  | 1.00 |  |  |
| Top quartile | 143 | 34349.47 | 4.16 | 0.86 (0.72, 1.04) | 0.120 |  | 0.97 (0.82, 1.22) | 0.979 |  |
| **Dietary score** |  |  |  |  |  |  |  |  | 25.40 (7.04,43.77) |
| < 2 ideal components | 575 | 115934.89 | 4.96 | 1.00 |  |  | 1.00 |  |  |
| ≥2 ideal components | 70 | 18141.53 | 3.86 | 0.78 (0.61, 1.00) | 0.046 |  | 0.88 (0.78,1.00) | 0.047 |  |
| Model 1: The impact of each lifestyle on the T2DM risk after adjusting other lifestyles.  Model 2: age, sex, family history of diabetes, family history of hypertension, prevalence of hypertension, use of anti-hypertensive medication, use of lipid-lowing medication, TG (mmol/L), HDL-C (mmol/L), and FPG (mmol/L). Individual lifestyle factors were mutually adjusted.  ^*^PAR% was calculated to estimate the percentage of T2DM that would have been prevented if all participants had been in low-risk behaviors.  CI, confidence interval; HR, hazard ratio; T2DM, type 2 diabetes mellitus. | | | | | | | | | |

| **Table S2 Adjusted HR of T2DM according to new HLS without alcohol behavior** | | | | | | | | |
| --- | --- | --- | --- | --- | --- | --- | --- | --- |
| **HLS** | Number of T2DM | Person-year | Incidence per 1000 person-years | Model 1 | |  | Model 2 | |
|  |  |  |  | HR (95% CI) | *P* |  | HR (95% CI) | *P* |
| 0-1 | 284 | 42814.01 | 6.63 | 1.00 | / |  | 1.00 | / |
| 2 | 282 | 62490.26 | 4.51 | 0.70(0.59,0.83) | <0.001 |  | 0.75(0.63,0.90) | 0.001 |
| 3-4 | 79 | 28772.15 | 2.75 | 0.42(0.33,0.55) | <0.001 |  | 0.43(0.33,0.56) | <0.001 |
| Per score | / | / | / | / | / |  | 0.70(0.63,0.77) | <0.001 |
| PAR% (%, 95% CI)^†^ | / | / | / | / | / |  | 43.54 (30.78,56.30) | / |
| Model 1: adjusted for age, gender, family history of diabetes, family history of hypertension, prevalence of hypertension, use of anti-hypertensive medication, use of lipid-lowing medication, and alcohol behavior.  Model 2: Model1+TG (mmol/L), HDL-C (mmol/L), and FPG (mmol/L).  ^†^PAR% was calculated to estimate the percentage of T2DM that would have been prevented if all participants had been in more than 4 healthy lifestyle behaviors.  HR, hazard ratio; HLS, healthy lifestyle scores; T2DM, type 2 diabetes mellitus; CI, confidence interval; PAR%, population-attributable risk percent. | | | | | | | | |

| **Table S3 Association of HLS with T2DM mediated by SUA after additionally adjusting for FPG** | | | | | |
| --- | --- | --- | --- | --- | --- |
| Effects | SUA^#^ (µmol/L) | |  | [Hyperuricemia](javascript:;)^†^ | |
|  | β (95% CI) | *P* |  | β (95% CI) | *P* |
| Total effects | -0.018 (-0.026, -0.012) | <0.001 |  | -0.018 (-0.027, -0.012) | <0.001 |
| Direct effects | -0.017 (-0.025, -0.010) | <0.001 |  | -0.017 (-0.025, -0.011) | <0.001 |
| Indirect effects | -0.001 (-0.002, -0.0006) | <0.001 |  | -0.001 (-0.003, -0.0006) | <0.001 |
| Proportion of mediation (%) ^*^ | 8.33 (3.56,14.93) | <0.001 |  | 7.15 (2.71,13.98) | <0.001 |
| ^#^The levels of SUA were nature log-transformed.  The model was adjusted for age, sex, family history of hypertension, use of anti- hypertensive medication, use of lipid-lowing medication, and FPG.  ^†^Hyperuricemia was defined as SUA ≥ 420 µmol/L for men and SUA ≥360 µmol/L for women.  ^*^1,000 bootstrap resampling.  HLS, healthy lifestyle scores; T2DM, type 2 diabetes mellitus; SUA, serum uric acid; FPG, fast plasma glucose; CI, confidence interval. | | | | | |

| **Table S4 Association of HLS with T2DM mediated by SUA stratified by sex** | | | | | | |
| --- | --- | --- | --- | --- | --- | --- |
| sex | Effects | SUA^#^(µmol/L) | |  | [Hyperuricemia](javascript:;)^†^ | |
|  |  | β (95% CI) | *P* |  | β (95% CI) | *P* |
| Male | Total effects | -0.019 (-0.036, -0.007) | <0.001 |  | -0.019 (-0.036, -0.007) | <0.001 |
|  | Direct effects | -0.018 (-0.034, -0.006) | <0.001 |  | -0.018 (-0.034, -0.006) | <0.001 |
|  | Indirect effects | -0.001 (-0.003,0.0001) | 0.08 |  | -0.001 (-0.002, -0.0001) | 0.04 |
|  | Proportion of mediation (%) * | 6.21 (-0.48,17.52) | 0.08 |  | 7.48 (0.52,15.51) | 0.04 |
| Female | Total effects | -0.032 (-0.052, -0.018) | <0.001 |  | -0.035 (-0.055, -0.020) | <0.001 |
|  | Direct effects | -0.027 (-0.047, -0.014) | <0.001 |  | -0.030 (-0.049, -0.016) | <0.001 |
|  | Indirect effects | -0.005 (-0.007, -0.003) | <0.001 |  | -0.005 (-0.007, -0.002) | <0.001 |
|  | Proportion of mediation (%) ^*^ | 14.39 (9.24,23.54) | <0.001 |  | 14.18 (6.37,21.97) | <0.001 |
| ^#^The levels of SUA were nature log-transformed.  The model was adjusted for age, family history of hypertension, use of anti-hypertensive medication, and use of lipid-lowing medication.  ^†^Hyperuricemia was defined as SUA ≥420 µmol/L for men and SUA ≥360 µmol/L for women.  ^*^1,000 bootstrap resampling.  HLS, healthy lifestyle scores; T2DM, type 2 diabetes mellitus; SUA, serum uric acid; CI, confidence interval. | | | | | | |

| **Table S5 Association of HLS with T2DM mediated by SUA** **stratified by age** | | | | | | |
| --- | --- | --- | --- | --- | --- | --- |
| Age (years) | Effects | SUA^#^ (µmol/L) | |  | [Hyperuricemia](javascript:;)^†^ | |
|  |  | β (95% CI) | *P* |  | β (95% CI) | *P* |
| < 60 | Total effects | -0.020 (-0.034, -0.009) | <0.001 |  | -0.020 (-0.034, -0.009) | <0.001 |
|  | Direct effects | -0.017 (-0.030, -0.007) | <0.001 |  | -0.018 (-0.031, -0.007) | <0.001 |
|  | Indirect effects | -0.003 (-0.004, -0.002) | <0.001 |  | -0.002 (-0.004, -0.0009) | <0.001 |
|  | Proportion of mediation (%) ^*^ | 13.63 (8.24,25.18) | <0.001 |  | 8.76 (5.11,20.04) | <0.001 |
| ≥ 60 | Total effects | -0.032 (-0.055, -0.014) | <0.001 |  | -0.032 (-0.055, -0.014) | <0.001 |
|  | Direct effects | -0.029 (-0.049, -0.011) | <0.001 |  | -0.029 (-0.051, -0.012) | <0.001 |
|  | Indirect effects | -0.003 (-0.005, -0.0006) | 0.04 |  | -0.003 (-0.005, -0.0006) | <0.001 |
|  | Proportion of mediation (%) ^*^ | 9.01 (1.75,21.36) | 0.04 |  | 8.49 (2.00,20.08) | <0.001 |
| ^#^The levels of SUA were nature log-transformed.  The model was adjusted for age, sex, family history of hypertension, use of anti-hypertensive medication, and use of lipid-lowing medication.  ^†^Hyperuricemia was defined as SUA ≥420 µmol/L for men and SUA ≥360 µmol/L for women.  ^*^1,000 bootstrap resampling.  HLS, healthy lifestyle scores; T2DM, type 2 diabetes mellitus; SUA, serum uric acid; CI, confidence interval. | | | | | | |
